# *“How can I do more?”* Cultural awareness training for hospital-based healthcare providers working with high Aboriginal caseload

**DOI:** 10.1186/s12909-020-02086-5

**Published:** 2020-05-29

**Authors:** Vicki Kerrigan, Nicole Lewis, Alan Cass, Marita Hefler, Anna P. Ralph

**Affiliations:** 1 Menzies School of Health Research, Charles Darwin University, PO Box 41096, Casuarina, Northern Territory 0811 Australia; 2 Department of Health, Northern Territory Government, GPO Box 2391, Darwin, NT 0801 Australia; 3 Royal Darwin Hospital, Darwin, Northern Territory 0811 Australia

**Keywords:** Aboriginal, Indigenous, cultural awareness, cultural safety, unconscious bias, hospital training

## Abstract

**Background:**

Aboriginal cultural awareness training aims to build a culturally responsive workforce, however research has found the training has limited impact on the health professional’s ability to provide culturally safe care. This study examined cultural awareness training feedback from healthcare professionals working with high Aboriginal patient caseloads in the Top End of the Northern Territory of Australia. The aim of the research was to assess the perception of training and the potential for expansion to better meet workforce needs.

**Methods:**

Audit and qualitative thematic analysis of cultural awareness training evaluation forms completed by course participants between March and October 2018. Course participants ranked seven teaching domains using five-point Likert scales (maximum summary score 35 points) and provided free-text feedback. Data were analysed using the Framework Method and assessed against Kirkpatrick’s training evaluation model. Cultural safety and decolonising philosophies shaped the approach.

**Results:**

621 participants attended 27 ACAP sessions during the study period. Evaluation forms were completed by 596 (96%). The mean overall assessment score provided was 34/35 points (standard deviation 1.0, range 31-35) indicating high levels of participant satisfaction. Analysis of 683 free text comments found participants wanted more cultural education, designed and delivered by local people, which provides an opportunity to consciously explore both Aboriginal and non-Aboriginal cultures (including self-reflection). Regarding the expansion of cultural education, four major areas requiring specific attention were identified: communication, kinship, history and professional relevance. A strength of this training was the authentic personal stories shared by local Aboriginal cultural educators, reflecting community experiences and attitudes. Criticism of the current model included that too much information was delivered in one day.

**Conclusions:**

Healthcare providers found cultural awareness training to be an invaluable entry point. Cultural education which elevates the Aboriginal health user’s experience and provides health professionals with an opportunity for critical self-reflection and practical solutions for common cross-cultural clinical encounters may improve the delivery of culturally safe care. We conclude that revised models of cultural education should be developed, tested and evaluated. This requires institutional support, and recognition that cultural education can contribute to addressing systemic racism.

## Background

Research from the colonised countries of Canada, Australia, New Zealand and the United States has found when Indigenous peoples access healthcare services they do not receive equal treatment [1–5]. Historically, health and medical practice in Australia was used as a tool of colonial control and the impact of a colonial history resulting in intergenerational trauma has been identified as a driver of Indigenous poor health [5–7]. In Australia, Aboriginal and Torres Strait Islander people access hospital services at 1.3 times the rate of other Australians and are admitted with a much greater burden of disease [8]. High mortality rates as a result of chronic disease, trauma and suicide have led to an unrelenting cycle of “sorry business” [9, 10]. “Sorry business” broadly refers to the cultural practices, including funerals, around death. Mainstream health services, operating according to biomedical norms and western concepts of wellbeing fail to deliver care which respects the cultural values and priorities of Aboriginal and Torres Strait Islander peoples [8, 11, 12]. Adverse consequences including death and absence of informed consent have been documented as a result of failures to deliver culturally safe care in Australian hospitals [13–17].

Regarding terminology, in reference to the First Nations people of Australia, Aboriginal or Aboriginal and Torres Strait Islander will be used. In discussing these issues, we acknowledge that Aboriginal and Torres Strait Islander peoples are “not culturally homogenous” [18] rather there is a myriad of distinct cultural groups with unique languages, knowledge systems and beliefs. However, as Behrendt states [18], Aboriginal and Torres Strait Islander peoples share a history of government dispossession and genocide which has influenced the collective psyche. For example, the government sanctioned forced removal of children from their families between 1910 and 1970, known as the “Stolen Generations”, continues to adversely impact on Aboriginal and Torres Strait Islander peoples [19].

The northernmost ‘Top End’ of Australia’s Northern Territory (NT), a remote sparsely populated and geographically large jurisdiction, offers a unique test case to explore the health professionals experience of cultural awareness training. Aboriginal people in the NT comprise 30% of the population, the highest proportion of any Australian state or territory [20]. The lifespan of people from remote parts of the NT is approximately 14 years less than the non-Indigenous population [21]. English is the language of the health system [22] however 60% of Aboriginal people in the NT speak one of the 100 Aboriginal languages spoken in the NT as their first language [23, 24]. At Royal Darwin Hospital (RDH), in the capital of the NT, 54% of patients identify as Aboriginal and most healthcare providers identify as non-Indigenous. Often they are from southern parts of Australia or overseas; 30% of Australia’s medical practitioners are trained overseas, the majority being from India, England and New Zealand. On commencing work in the NT, new staff may experience culture shock, or even “a sense of ‘hopelessness”, as they are often unprepared for the unique medical and complex cultural environment [22, 25]. To assist in this regard, healthcare providers are offered Aboriginal cultural awareness training. However, a previous study at RDH found staff appreciated cultural awareness training but were dissatisfied with its scope which failed to address racism (unconscious and institutional) [25].

The theories behind cultural education have been extensively explored by scholars [26] however there is a paucity of Australian-specific evidence regarding the practical educational strategies which are effective in improving quality of care for Aboriginal and Torres Strait Islander Australians [22, 27, 28]. There are some who argue that all forms of cross-cultural education should be discontinued until there is evidence it is necessary [26]. However, the lack of evidence may be attributable to the difficulties associated with connecting the impact of training to improvement in service due to the range of variables that determine patient outcomes. The infancy of this field is also apparent through the lack of consensus around terminology [3, 6, 29]. Terms such as cultural awareness, cultural competence and cultural safety (to name a few) have been used interchangeably. However, each are slightly different and some scholars argue these terms exist along a cultural continuum, indicated by the order above [2, 22, 27, 30], with cultural awareness at the beginning. For necessary context, a brief overview of terms is outlined. Cultural awareness training aims to educate non Aboriginal people about aspects of Aboriginal beliefs and practices however cultural awareness training can reinforce negative stereotyping [1, 26, 31]; it has been likened to “going to a museum but then you go home” [22]. Cultural competence is more than awareness, it focuses on the capacity of the health system to integrate culture into the delivery of health services [29]. Cultural competency interventions have been found to improve client/practitioner relationships which consequently increased health service access [32]. In contrast to cultural awareness and competency, cultural safety is more challenging as it requires critical examination of the self and the broader health system [29]. While some argue cultural safety is part of a continuum of learning (awareness, then competence, then safety) we subscribe to the idea that cultural safety actually requires a “paradigm shift” [33]. Cultural safety is embedded in critical theory and social justice concepts, it requires hegemonic individuals and organisations to self-reflect and critique the power structures which contribute to maintaining health inequities [6, 34]. Overall scholars agree that further research is required to explore the impact of cultural education on clinical practice and racially-based healthcare disparities [32]. In the meantime, cultural awareness training continues to be developed and delivered by health organisations.

This research is embedded in the ‘Communicate’ study [35]. We are working with Aboriginal health service users, Aboriginal interpreters and medical professionals to explore options to address the intercultural communication barriers between health providers and Aboriginal patients in the Top End. Poor patient-provider communication adversely impacts on the delivery of equitable care [6] and has been identified as one of the most common ways clients experience racism in the health setting [36]. Communication is shaped by unconscious ideas which can be difficult to acknowledge and control, including stereotypes and negative attitudes which contribute to racial/ethnic disparities [37]. A contributing factor to negative stereotyping is a lack of cultural knowledge. Research from North America, Australia and New Zealand found that well intentioned cultural awareness training, focusing on Aboriginal cultures with little consideration of the broader health service and dominant culture, does little to address the health inequities experienced by racial and ethnic minorities [26–28, 31]. Assessing the impact of cultural awareness training on patient outcomes is difficult to track. For this reason, the aim of this research was to assess the health care professionals’ (clinical and non-clinical) perceptions of cultural awareness training after attendance, to identify strengths and weaknesses and the potential for expansion according to workforce needs. To the best of our knowledge this is the first time such research has been undertaken in this jurisdiction.

## Methods

### Study setting and context

In the tropical far north of Australia, the Top End Health Service (TEHS) manages three hospitals: RDH, Katherine and Gove Hospital, and 57 remote health clinics. TEHS provides over 100,000 episodes of inpatient care annually and employs approximately 4260 full time equivalent staff [38]. During the study period, TEHS offered a one-day (7 hours), face-to-face Aboriginal Cultural Awareness Program (ACAP) which was delivered by one trainer from the NT Department of Health. The trainer (second author) NL resides in Darwin and her family come from Gurindji country or what is now referred to as the Victoria River area of the NT. When NL was unavailable, the course was delivered by another experienced cultural educator (a member of the Muran clan) with family connections through north western Arnhem Land and Kakadu in the NT. A TEHS Key Performance Indicator states 100% of staff must have participated in cultural awareness training before June 2017 [39]. By September 2018, approximately 30% of staff had completed the training [40]. Cultural awareness training became mandatory for all new staff in 2018 [41] however existing staff do not have protected time, away from existing duties, to attend training during work hours.

In addition to the full day ACAP, some TEHS employees deliver cultural education including an abridged two-hour ACAP session [39]. This paper only reports on the evaluation of the full day face to face ACAP training.

### Design

Our research is shaped by the philosophical underpinnings of cultural safety which recognises that the onus for change resides with hegemonic individuals and institutions [2]. Healthcare providers, who critically reflect on the culture of the health care system, and their position within the system, are well placed to advocate for change from the inside [22]. This aligns with the philosophical approach of community development practitioners who theorise that for sustainable positive change to occur the institutions and values, which perpetuate entrenched inequalities, must change [42]. The research was also influenced by decolonising principles [43, 44]: NL’s (second author) lived experience as an Aboriginal health care user, a patient escort and a cultural educator, contributed to the data collection, analysis and discussion.

One day face-to-face ACAP sessions were delivered between March and October 2018. The ACAP covers the five workshop themes commonly canvassed in cultural awareness workshops in North America and Oceania: interaction approaches (communication), belief systems, historical matters, discrimination and organisational issues [26]. Training priorities are guided by the lived experience of cultural educators, the Ottawa Charter for Health Promotion [45] and various NT government reports including the NT Department of Health Strategic Plan which aims to “improve Aboriginal health outcomes” and “build a highly skilled and culturally responsive workforce” [46] to meet community needs.

### Data and analyses

A mixed methods approach was used to collect and analyse data. Anonymous evaluation forms were completed by participants at the conclusion of each training session. Feedback was entered into an excel spreadsheet by NL for analysis and the de-identified data collated for each training session was provided to the auditor (VK) for analysis.

#### Quantitative data

Using a five-point Likert scale, participants ranked the quality of training in five key domains: introduction to culture; introduction to language, communication and working with interpreters; introduction to kinship system; concept of Aboriginal well-being; and Indigenous history. A final score out of 35 was generated. The mean score (out of a maximum 5 points) assigned for each of the seven topic areas was calculated, as was the overall summary score for the course (out of a maximum 35 points).

#### Qualitative data

Qualitative (free-text) options were included in the form to allow participants to provide feedback including suggested topics for future training. The Framework Method, commonly used in health research and well suited to the thematic analysis of data sets which cover similar issues [47], was used to analyse data. Initial coding using NVIVO 12 software was conducted by VK. Themes were refined through an iterative process with VK, NL, MH and AR. As the Framework Method can be applied to various methodological approaches we also drew on phenomenological methods to code “significant statements” [48]. Statements are presented as direct quotes to ensure participant experience is accurately portrayed.

Finally, Kirkpatrick’s training evaluation model [49] was chosen as the appropriate explanatory framework to assess and interpret results in the discussion. Kirkpatrick’s model evaluates training across four levels: 1) learner reactions; 2) learning; 3) on the job behavior change and; 4) observable organisational results [50].

## Results

### Participation

Six hundred twenty-one TEHS staff from Darwin, Katherine and Gove attended 27 ACAP sessions from March to October 2018. Demographic details such as age/gender/ethnicity/professional discipline were not collected on evaluation forms however some participants shared such information in free text comments. Participants included: experienced health professionals plus new recruits; Indigenous and non-Indigenous healthcare providers from Darwin, other regions of Australia and international healthcare graduates.

### Ranking of teaching domains

**Fig. 1:**
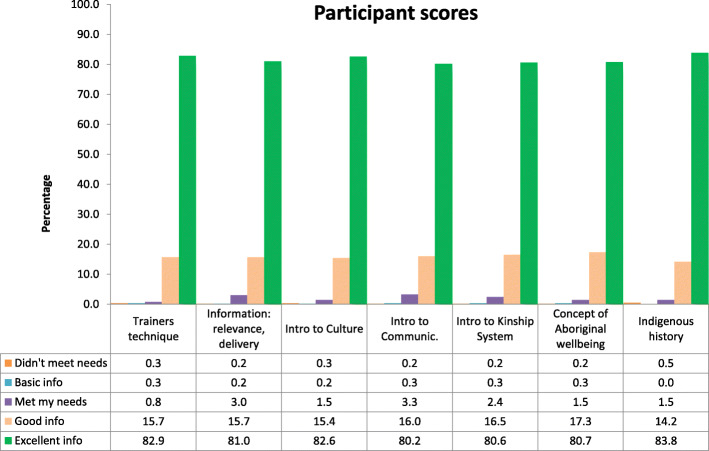
Participant rating of Aboriginal Cultural Awareness Program training day

Five hundred ninety-six evaluation forms were collected (completion rate 96.0%). The mean overall assessment score provided by participants for the 27 ACAP sessions was 34 points (standard deviation 1.0, range 31-35). Scores across the seven individual topic areas were all high (Figure 1) which revealed the training provided good to excellent information across all topics. Considering the high numerical scores with largely consistent responses across all domains, the authors decided to concentrate analysis on the free text which provided a richer and more diverse data source.

#### Thematic analyses

Six hundred eighty-three free text comments which varied in length from single word answers to a 74-word paragraph were analysed. An evaluation of learner reactions revealed participants were satisfied with the ACAP describing the course as *“vitally important”* and stating training should be mandatory for NT government employees*.* One participant had “*completed over 100 hours [of] cultural training*” and stated that “*this was the first strengths-based presentation”.* Presenters were praised for their engaged and *“passionate*” approach with one participant exclaiming: *“this is the best ACAP I have attended in 44 years working in the health system"*. Teaching resources including PowerPoint presentations and videos were valued. The following quotes indicate participants from different cultural backgrounds acknowledged lessons learned:

*“A very interesting day of learning Aboriginal culture. Even for an Indigenous person my eyes have been opened to so much I did not know.”* – Participant feedback

*“So helpful. I am a kiwi, relatively ignorant- I feel more informed but astounded this is the circumstances in Australia.”* – Participant feedback

Only one comment stated the training was extraneous: *“waste of my time and hospital resources”.* Other complaints referred to lack of tea and coffee making facilities and training logistics including course duration and location.

The three key findings were: 1) participants expressed a desire for more cultural education; 2) designed and delivered by local people; 3) which provides an opportunity to consciously explore both Aboriginal and non-Aboriginal cultures (including self-reflection). As the request to expand cultural education was multifaceted, we identified four major areas requiring specific attention: communication, kinship, history and professional relevance.

##### More cultural education*: “How can I do more?”*

Learning about and understanding Aboriginal culture was recognised as “*a continual learning process for staff”.* Many participants stated too much information was delivered in one day and requested the training be expanded to avoid feeling overwhelmed, and to create opportunities for discussion and self-reflection. Suggestions included delivering the training over two days, follow-up training every three to six months, and online modules to refresh learning, including videos which portray common clinical scenarios.

“For a person who comes from the south*, this education needs to be more in depth to ensure that I/we are better culturally (sic) *and ensure that nurses like me can serve the community in the best way at all times of care.”* – Participant feedback (*south refers to southern Australian states, where the proportion of Aboriginal and Torres Strait Islander people is lower and the cultural context different).

The four areas requiring further training will be explored below:

Communication: Intercultural communication training was identified as the greatest area of need. More in-depth training on working with Aboriginal interpreters was requested, as well as practical advice on how to communicate, verbally and non-verbally, in the clinical setting if an interpreter was unavailable.

*“Communication methods, socially acceptable behaviours, more understanding of cultural norms that can affect clinical practice e.g. male and female interaction rules.” –* Participant feedback

Participants were also interested in learning key phrases or greetings from commonly spoken Aboriginal languages in the region. At Gove District Hospital, where patients mostly speak Yolŋu Matha, staff requested lessons in relevant dialects. Participants valued new knowledge around how commonly used terms in medicine may be misinterpreted. For example, the word “deadly” is often used by Aboriginal peoples to mean fantastic, not fatal.

Kinship: An overview of the Aboriginal kinship system was appreciated with some participants stating the lessons learned can be applied to clinical work. However due to the complexity of kinship, many found information was *“overwhelming”* and “*difficult to follow”*. A stand-alone course on the structure and responsibilities of social relationships was requested so healthcare providers can better understand *“why we must do things”* when working with Aboriginal patients.

History: The importance of understanding the history of colonised Australia was recognised and more information about the Stolen Generations was requested. Teachings on history were considered vital by those indicating they had recently arrived in Australia and also Australian-born health professionals.

*“Indigenous history: quite confronting, we don't learn about this in school”* –Participant feedback

Relevant to health profession: Some non-clinical staff suggested training could be reduced to a half day, or less, for non-clinicians. Health professionals with patient contact requested service specific information. How to gain informed consent, including identifying the appropriate guardian for a child, was repeatedly mentioned as an area of concern. Practical advice on how to verify the identity of patients unaware of their date of birth was also requested. Clinicians sought more training applicable to their specialties: emergency department, palliative care, midwifery, drug and alcohol, renal, mental health and paediatrics.

*“I would love to learn more about Aboriginal and Torres Strait Island culture if there was also a specific discipline presentation, for example ‘Cultural Awareness for Aboriginal and Torres Strait Islanders in Maternity’”* – Participant feedback

##### Designed and delivered by local people: *“Great use of personal stories”*

An abundance of appreciative feedback related to the personal stories and *“real life anecdotes*” shared by trainers. The personal stories elevated the *“Aboriginal person’s experience*” creating an opportunity for participants to empathise and thereby relate learnings to clinical work.

*“Enjoyed* (trainer’s name) *personal stories related to their experiences as an Aboriginal person navigating the 'western' system.”* – Participant feedback

Local information relating to the NT was new information for many. Site specific information about working with, and the pressures faced by, Aboriginal Liaison Officers (ALO) and Aboriginal Health Practitioners (AHP) in the Top End was also beneficial.

*“Didn’t realise strain load on ALOs and AHPs within the hospital system”* – Participant feedback

##### Culture: *“Would be great to meet some local Elders to share local issues”*

A desire to learn more about the diversity of Aboriginal communities through interactions with Elders and emerging leaders was also expressed. The need for more education about Aboriginal concepts of wellbeing and cultural practices, including the use of bush medicine and traditional healers and how that may intersect with hospital care, was a recognised knowledge gap. Participants expressed a desire to know more about men’s and women’s business; sorry business protocols; ceremonies; tribal law and payback. Participants reasoned that if health professionals had a better understanding of cultural obligations, they may be able to reduce the number of patients who “take their own leave” from hospital.

*“Sorry business protocols - Relevant to patients taking their own leave* [from] *hospital.” –* Participant feedback

Cultural safety training to address unconscious bias and institutional racism was also requested. Participants noted the need to reflect on their own cultural background, privilege and power:

*“A session about our own concept of culture i.e. we have ‘culture’ and this affects our response to other cultures.” -*Participant feedback

## Discussion

This study builds on previous research identifying the need to improve cultural awareness training [25, 51] because health professionals need “to have a degree of knowledge and understanding of other cultures” [6]. As a result of low ACAP attendance, assumptions exist that healthcare providers do not value the training but we found those who attend recognise cultural awareness is an important foundation. The discussion will be divided into two parts. Firstly, Kirkpatrick’s evaluation model will be used as a framework to interpret findings, and secondly, options to expand cultural education will be explored.

### ACAP evaluated according to Kirkpatrick’s model

#### Level 1: Reaction

Most participants enjoyed the training as indicated by the high numerical scores displayed in Figure 1 and the free text comments outlined. We assert a major contributor to the positive feedback was due to the trainer’s enhancing the set curriculum with personal stories as users of the health service and active members of the local community. We recognize this as a strength of the training, discussed in more detail below.

#### Level 2: Learning

Learning about the historical determinants of health relating to Aboriginal peoples in the Northern Territory and Australia was a revelation to both Australian and overseas educated participants. Participants appreciated knowledge which related to the clinical setting: insights into how the kinship system may impact patient care and learning Aboriginal English [52] terms such as “deadly” was valued. Top End specific information about the role of ALO’s and AHP’s who juggle cultural obligations alongside incompatible expectations of non-Indigenous colleagues often in the face of discrimination, lack of professional support and chronic illness [53, 54] was considered valuable.

#### Level 3: On the job behaviour change

Due to the nature of the evaluation it was impossible to assess if the ACAP resulted in behavior change however from the data we can surmise that health professionals aspire to transfer learnings to the workplace especially if training is expanded.

#### Level 4: Observable organisational results

The authors determined evaluating level 4 is beyond the scope of this data. Kirkpatrick states, it is “difficult, if not impossible” to evaluate the impact of training on an organisation due to an inability to separate the variables which could be attributed to training or other factors [49]. Researchers working on the aforementioned ‘Communicate’ study [35] are undertaking projects which may address outstanding questions regarding the impact of cultural education on service delivery, staff turnover and operational costs.

### Expanding cultural education for workforce needs

Aligning with previous research [55], participants recognised that insufficient cultural education is a barrier to providing culturally appropriate care. Disrespectful communication coupled with institutionalised racism and collective past experience, can result in an individual or family being unwilling to engage with a system they don’t trust [39]. ACAP participants want more cultural education, designed and delivered by local people, which provides an opportunity to learn about Aboriginal cultures and critically explore the interface with non-Aboriginal cultures. Specific topics, which should be expanded to meet workforce needs were: communication, kinship and history. Participants were also adamant that training should be tailored to professions. The communication style of health professionals has previously been described as “an apparatus of colonisation” used to control Indigenous peoples [56]. Conversely, good interpersonal communication contributes to improved health outcomes, consumer engagement and adherence [51, 57]. Participants recognised that learning key phrases in local Aboriginal languages was an important component of building patient-provider rapport and may create a gateway to levelling out the power differential. Participants requested more in-depth information about kinship; however, this may be difficult for cultural educators because specific knowledge must be shared by “traditional knowledge holders” from the various Aboriginal nations [58]. However, understanding that kinship relationships impact on how Aboriginal people navigate the health system is crucial [59]. McBain-Rigg and Veitch assert that for some Aboriginal patients, health issues are framed in relation to family and community connections and the importance of personal relationships extends to encounters with health professionals. Patient- provider relationships based on trust and respect are a necessary part of good healthcare; yet for the health professional, trained to focus on task-based issues, this approach is an anomaly [59, 60]. This cultural difference needs to be understood by the health professional if they are to deliver culturally safe patient centred care. It is crucial health professionals, both Australian and overseas born, reflect on the history of colonisation to better understand the circumstances which lead to individuals requiring their service [22]. The impact of the ‘Stolen Generation’, for example, contributes to the ongoing impairment of the health and wellbeing of Aboriginal and Torres Strait Islander peoples [53]. Cultural safety advocates for the demystification of colonial history [2]. Finally, it was clear that a one size fits all approach to cultural awareness training should be reconsidered. Shepherd [26] argues training does little to assist the medical professional to practically address the “nuances typical of a cross cultural encounter” in the clinical setting. Recognising the complexities of the dynamic hospital environment, ACAP participants requested cultural education tailored to roles; doctors, nurses and allied health professionals need knowledge and skills specific to their clinical tasks [3]. Cultural education which inspires non-clinical staff to critically think about the structures in which they work is also imperative: service planners and policy makers have the power to create systems which support the delivery of culturally safe care.

### The future of cultural education in the Top End

The concept of culture is central to cultural education [61]. Many healthcare users have a different worldview to the western biomedical approach which underpins mainstream healthcare delivery [32]. An awareness of differing worldviews [62, 63] and the ability to self-reflect on one’s own culture and the broader culture of the healthcare system is required by the culturally safe health professional [64]. Training needs to expand beyond the superficial “museum approach” [26] towards cultural safety which creates opportunities for health professionals to reflect on interpersonal power differences between provider and patient and also the policies and systems which contribute to inequities in health care [6]. Concordant with Curtis et al [6], this research revealed that health professionals recognise cultural competency does not have an endpoint but instead employees should be engaged repeatedly [65] as is the case with mandatory training including annual fire safety and biennial hand hygiene training. The ‘one-shot’ cultural awareness classroom-based training is unlikely to influence behaviour in clinical contexts as health professionals are better able to form new habits in situ through continuous practice [1]. In the high-pressure hospital environment, clinicians are “acutely aware of the constraints these environments have on skills gleaned from short professional development exercises” [26]. A communication training intervention delivered in a European hospital found doctors were able to quickly apply lessons learned and as a result, patient-centered care improved [66].

TEHS is working to rectify low attendance rates by exploring ways to redesign the delivery of cultural awareness training. In 2018, RDH made the full day face to face ACAP mandatory for all new staff [41]. Whilst upfront orientation prior to commencing work in a new location is vital, we contend if culturally safe patient-centered care is to be delivered, ongoing opportunities are required to address questions arising from clinical experiences. For example, after attending ACAP, experienced Top End health professionals expressed a desire to learn more about the social and cultural obligations of Aboriginal patients, including “sorry business” protocols. Greater understanding of such obligations and protocols may contribute to reduced self-discharge rates. Patients who discharge against medical advice (DAMA) from NT hospitals is a substantial problem and adverse outcomes include suspension of medical treatment, health costs to individuals, negative impacts on staff morale and high system costs [67]. Aboriginal patients are more likely to take their own leave from hospitals and this is viewed “as an indirect measure of cultural safety” as it indicates the extent to which hospitals are responsive to the needs of Aboriginal patients [68]. Further research is required to explore if a redeveloped cultural education package could improve cultural safety and reduce DAMA rates. This research may go some way to addressing Kirkpatrick’s elusive level 4: observable organisational results.

We acknowledge employment of more health professionals who identify as Aboriginal would improve the delivery of culturally safe healthcare. There are 100,000 medical practitioners registered in Australia and 0.5% identify as Aboriginal or Torres Strait Islander [69]. It would be unfair to place the burden of improving health outcomes on Aboriginal health professionals alone [11, 22]. We believe it is the responsibility of the majority of the workforce to examine and adapt their practices to improve health outcomes of Aboriginal people [22]. The Australian healthcare workforce is dominated by non-Indigenous Australians and relies on overseas trained professionals [22]; 31.5% of medical practitioners gained qualifications overseas and from that cohort India were the largest group, followed by England and New Zealand [69]. Health professionals, Australian and internationally trained, who have had limited relations with Aboriginal people may bring preconceived stereotypes, often shaped by negative media reports, to their roles [30]. Negative stereotyping of Aboriginal patients has been identified by the NT Health Department as a contributor to “people feeling disrespected or not receiving the best care possible” [70]. The well-intentioned cultural awareness training commonly delivered in Australian hospitals can reinforce negative stereotypes [1, 31] as it may perpetuate “othering” of Aboriginal people. Instead, stereotypes associated with minorities (unconscious bias) may be addressed by providing counter stereotypical stimuli such as increasing opportunities for positive experiences [1] or engaging Aboriginal leaders to share “authentic stories with strong messages about the health needs of Aboriginal people” [71]. In this ACAP, the possibility of ‘othering’ was diminished by the Aboriginal trainer’s emotive and relatable stories which expanded the set curriculum. The personal stories exposed differing ontologies and created an opportunity for the healthcare provider to examine their beliefs, position of power and the impact these may have on others [2]. In spite of funding constraints [72], opportunities to employ more cultural educators and to collaborate with Aboriginal Elders to document and share stories with staff to counter racialised assumptions and stimulate self-reflection should be explored. Regular reflexive learning circles in which health providers share experiences to troubleshoot common intercultural scenarios may be a worthwhile consideration. In a non-judgmental setting, with cultural educators as facilitators, actionable strategies coupled with personalised feedback regarding bias may result in attitude and behaviour change [65]. Research is required to evaluate cost savings that could be achieved by investing in cultural education to support the development of a culturally safe workforce.

Accommodating cultural education into the busy clinical schedule of healthcare providers, working in an under resourced health service, requires organisational flexibility and commitment. Options worthy of consideration include protected (paid) time during clinical placements or development of engaging after-hours opportunities. Incorporating cultural education into work hours signals it is valued by the organisation [61] and is not a symbolic tick-box exercise [26]. It could also address the need for observable organisational results as noted by Kirkpatrick’s Level 4 [49] including increasing attendance rates. Institutions which do not prioritise staff attendance at cultural education may be accused of institutional racism, whereas a culturally safe organization is committed to ensuring staff attend training [6]. Podcasts are recognised as useful tools in medical education as the flexible format can deliver “high yield information in a short time” [73, 74]. In contrast to video or online training, podcasts can easily be integrated into busy schedules. Considering the reliance on online training modules, it is relevant to note that audio has been found to be more effective than text based learning [75].

A potential limitation of this study is selection bias; those who attend a whole day ACAP may be more interested in cultural awareness than peers who do not attend. The auditor did not have access to the original evaluation forms; transcribing errors may have occurred between paper forms and the electronic database. However, data entry was completed by an NT Health Department employee experienced in data entry, so the risk of systematic error is low. We also recognise the findings of this project may not be generalisable to other health jurisdictions due to different social, cultural and historical contexts. We deliver this research with a cautionary note: whilst we have revealed opportunities to re-develop cultural training according to workforce needs, in order to meet community priorities all cultural education must be developed in consultation with Aboriginal educators and community leaders.

## Conclusion

Cultural awareness training is a phenomenon, which continues to be developed and delivered by health organisations despite the extensive academic critiques. Our research found the ACAP delivered to Top End healthcare providers is an invaluable entry point revealing important information which stimulates further enquiry. We assert a key reason participants valued this training was because of the authentic personal stories shared by local Aboriginal educators. Healthcare providers require ongoing cultural education, localised to the region where they are working, which can be applied to the clinical environment. It is crucial the education creates opportunities to address unconscious bias and institutional racism through critical self-reflection. A suite of initiatives, including engaging relevant cultural education, are required to improve the delivery of culturally safe care for Aboriginal and Torres Strait Islander healthcare users. Further research is required to examine cost effective ways to expand cultural education which addresses the needs of staff, the organization, the patient and their family.

## Data Availability

The data that support the findings of this study are the property of the Northern Territory Department of Health; restrictions apply to the availability of these data, which were used under license for the current study. Data are however available from the authors upon reasonable request and with permission of NT Department of Health.

## Abbreaviations

ALO: Aboriginal Liaison Officer
AHP: Aboriginal Health Practitioner
ACAP: Aboriginal Cultural Awareness Program
DAMA: Discharge Against Medical Advice
NT: Northern Territory
RDH: Royal Darwin Hospital
TEHS: Top End Health Service
